# Improving STD testing behavior among high-risk young adults by offering STD testing at a vocational school

**DOI:** 10.1186/1471-2458-11-750

**Published:** 2011-09-30

**Authors:** Laura WL Spauwen, Christian JPA Hoebe, Elfi EHG Brouwers, Nicole HTM Dukers-Muijrers

**Affiliations:** 1 Department of Infectious Diseases, South Limburg Public Health Service, Geleen, The Netherlands; 2 Department of Medical Microbiology, School of Public Health and Primary Care (CAPHRI), Maastricht University Medical Center (MUMC+), Maastricht, The Netherlands

## Abstract

**Background:**

Chlamydia trachomatis infection (CT) is the most prevalent bacterial STD. Sexually active adolescents and young adults are the main risk group for CT. However, STD testing rates in this group are low since exposed individuals may not feel at risk, owing-at least in part-to the infection's largely asymptomatic nature. Designing new testing environments that are more appealing to young people who are most at risk of acquiring chlamydia can be an important strategy to improve overall testing rates. Here we evaluate the effect of a school-based sexual health program conducted among vocational school students, aiming to obtain better access for counseling and enhance students' STD testing behavior.

**Methods:**

Adolescents (median age 19 years) attending a large vocational school were provided with sexual health education. Students filled in a questionnaire measuring CT risk and were offered STD testing. Using univariate and multivariate analysis, we assessed differences between men and women in STD-related risk behavior, sexual problems, CT testing behavior and determinants of CT testing behavior.

**Results:**

Of 345 participants, 70% were female. Of the 287 sexually active students, 75% were at high risk for CT; one third of women reported sexual problems. Of sexually active participants, 61% provided a self-administered specimen for STD testing. Independent determinants for testing included STD related symptoms and no condom use. All CT diagnoses were in the high-CT-risk group. In the high-risk group, STD testing showed an increased uptake, from 27% (previous self-reported test) to 65% (current test). CT prevalence was 5.7%.

**Conclusions:**

Vocational school students are a target population for versatile sexual health prevention. When provided with CT testing facilities and education, self selection mechanisms seemed to increase CT testing rate dramatically in this high-CT-risk population expressing sexual problems. Considering the relative ease of testing and treating large numbers of young adults, offering tests at a vocational school is feasible in reaching adolescents for STD screening. Although cost-effectiveness remains an issue counseling is effective in increasing test rates.

## Background

Sexually transmitted diseases (STD) pose a major public health problem. Chlamydia trachomatis infection (CT) is the most prevalent bacterial STD; its prevalence has increased in many countries, including The Netherlands [1,2]. Chlamydia infection can cause significant morbidity, particularly in women: up to two thirds of cases of tubal infertility and one third of cases of ectopic pregnancy may be attributable to chlamydia infection [3]. Sexually active adolescents and young adults are the main CT risk group [4-7] and those with lower and intermediate levels of education are especially at risk for CT [8]. Several studies demonstrated a high CT prevalence among students attending schools for low and intermediate education (vocational schools) [5,7,9-14], including a Dutch study showing a CT prevalence of 24.5% in vocational schools compared to 4.5% in general in the same study area, and 2.3% nationwide among Dutch sexually active 15-29-year olds [9,15]. To trace more infections and reduce transmission within the population, increasing the testing rate is of tantamount importance, especially in those who are at high risk. Interrupting CT's route of transmission by identifying and treating patients is an essential intervention in the prevention of this STD.

Although current sampling methods, i.e., self-taken vaginal swab (SVS) and first catch urine (FCU), are easy to perform and well accepted [16], and treatment is simple (one dose of 1000 mg azythromycin), reluctance of at risk populations to attend appropriate care and fear of invasive gynecologic examination hamper effective STD control [16]. While adolescents and young adults may not feel at risk since CT infections are often asymptomatic (approximately 70% in women and 50% in men) [10,13,17,18], they have a high prevalence of unsafe sex and low STD testing rates [19]. Only 8% of all consultations of STD centers are in the group of 15-19 year old attendees [8]. Not only the high prevalence of STD, but also the prevalences of other sexual health issues among young adults in the Netherlands, like increase of teenage pregnancies and high rates of sexual problems, are alarming. In a nationwide study among young adults, many sexual problems are reported [19]. In the Netherlands, public health services coordinate STD clinics and are responsible for STD control and sexual health counseling. Attention for sexual problems is increasing, and STD clinics recently extended their services to a new area called 'Sense' where clients may seek advice for non-STD related sexual problems.

A recent study among sexually active vocational school students in the Netherlands found that intention to get tested and perceived susceptibility to infection was very low [20]. Given the low rate of chlamydia testing among young people [8,19], identifying alternate settings that are more attractive to young people who are most at risk of acquiring chlamydia is likely to be an important strategy for increasing overall testing rates [21]. In several countries in Europe, including the Netherlands, different large scale screening programs have been subject to evaluation. The body of evidence for the effectiveness of population screening programs is limited [22,23], and participation rates are low [24,25]. The results of a systematic, Internet-based CT screening program in the Netherlands showed an overall participation rate of 16% in the first year [24]. This overall effective testing rate seems low [26], underlining the need to explore other screening strategies.

There is urgent need to enhance the accessibility of sexual health services for adolescents in order to improve participation rates and sexual health in this group. Here we evaluate the effect of a school-based sexual health program conducted among vocational school students, aiming to obtain better access for counseling and enhance students' STD testing behavior.

## Methods

### Study population

The study population included students of the largest vocational school in the South of the Netherlands, covering four vocational branches (administration and business, health and well-being, technology and hotel and catering industry and tourism), attended by more than 9000 students mostly aged 18 to 20 years. Eighteen of 240 classes (n = 348 students) from three branches (health and well-being; hotel and catering industry and tourism; technology) were approached for participation.

### Procedure

All 348 students of the 18 classes, each including an average of 19 students, first received a 45-minute presentation by a trained nurse, concerning STD in general and CT and risk behavior in particular. Of these students 99% signed for informed consent, yielding 345 study participants. All participants were asked to fill in a questionnaire. Subjects were asked to submit a self-administered specimen for STD testing, after receiving instructions on how to take a self-administered vaginal swab (SVS) or first catch urine (FCU) (for men only). Prior studies showed that SVS were equivalent in sensitivity and reliability to traditional endocervical swab specimens and more sensitive and reliable than self-obtained urogenital specimen for the detection of CT and GO [16]. While the sensitivity of FCU may be somewhat lower than the sensitivity of SVS, data show that sensitivity may be no less than 87.7% [27]. This method is also widely used in both individual and population screening studies, and is now the CDC-recommended method of sampling for nucleic acid based testing [28]. Both SVS and FCU are used as standard STD clinic tests.

Submitting a specimen was voluntary. Students were free to take the test at home and return specimens the next day. To avoid peer pressure, all students were asked to return materials in the given coded envelope and drop it in a closed box installed at the school's bathrooms.

This study was approved by the Medical Ethical Committee of the University of Maastricht (MEC 07-3-089) and registered in the Dutch National Trial Register (NTR1410). Originally the study was conducted as a randomized controlled trial which was discontinued for lack of effect. Data from both the intervention (n = 186) and control group (n = 159) were pooled in current analyses since there was no intervention effect (OR: 0.731, p = 0.150), and intervention and control groups were largely similar, except that participants in the intervention group were somewhat older (41% versus 27% were 20 years or older, p < 0.01). In short, the intervention consisted of a tailored CT test advice which was the result of a personal score on a self-triage card. This card was provided before handing over the self-test materials but after students had had the sexual health education and had filled in the questionnaire. The questions on the triage-card, scoring method, and CT test advice was based on a Dutch population study which found that chlamydial infection was associated with high urbanization, young age, ethnicity (Surinamese/Antillean), low/intermediate education, multiple lifetime partners, a new sexual partner in the previous two months, no condom use at last sexual contact and symptoms of (post)coital bleeding in women and frequent urination in men [29]. A risk score based on an algorithm from these findings has the potential to guide individuals in their choice of participation when offered chlamydia screening [29]. Based on the score, the intervention group received a positive or negative CT test advice which was printed on the card. However, all students were free to submit a sample, even if they had a negative test advice.

### Laboratory

The SVS and FCU samples were tested for the presence of CT and Neisseria gonorrhea (NG) by Nucleic Acid Amplification Tests (NAAT) with internal inhibition control: Strand Displacement Amplification assay (SDA) of Becton Dickinson Probe Tec ET system, Maryland, USA. Of note, none of the samples were positive for NG.

### Feedback of STD test results and treatment

For feedback of testing results, standard STD center procedures were used, i.e., subjects were requested to provide a phone number or an e-mail address. Negative test results were returned in most cases by SMS or, in some cases, by e-mail. In case of a positive test result, STD nurses telephoned, offering treatment and partner notification. All CT-positive students were notified and were treated with one oral dose of 1000 mg azythromycin.

### Questionnaire and statistical analysis

The questionnaire comprised the same items as the self-triage card supplemented with questions about partners, sexual behavior, drug use and sexual problems for adjustment of potential confounders. For participants who were sexually active a risk score was calculated from seven questions in the questionnaire. High risk was defined by an algorithm based on the association of chlamydial infection with high urbanization, young age, ethnicity (Surinamese/Antillean), low/intermediate education, multiple lifetime partners, a new sexual partner in the previous two months, no condom use at last sexual contact and symptoms of (post)coital bleeding in women and frequent urination in men. This dichotomous score (high versus low risk) has been shown to be predictive in discriminating CT-infected from CT-uninfected Dutch adults [29]. Predictive value based on a sum score of 6 was 3.5% [29].

Sexual mixing and concurrency of partners over the preceding six months were included as variables, based on self-reported information provided by the respondents. Partnerships that involve mixing behavior, as well as concurrent partners, have the potential to rapidly accelerate the spread of STD in a population [30]. Mixing by age was grouped into high and lower risk for STD; for women, high risk to acquire an STD was defined by at least one older partner, with a minimum age difference of one year; for men, high risk for STD transmission was defined by at least one younger partner, with a minimum age difference of one year [7,31]. A variable concerning sexual orientation was included, based on self-reported same-sex activities (at least one reported same-sex partner) and opposite-sex activities. Partnerships were classified as concurrent if participants reported at least two partners simultaneously. Rates of reported sexual problems, risk behavior and CT testing were calculated and differences between men and women were assessed using chi-square testing. Sexual problems were categorized into three groups: problems with sex (problems with sex in general, problems with desire, arousal or orgasm, pain), problems related to teenage pregnancy (worried about being pregnant, use of morning-after pill, induced abortion, pregnancy test, pregnancy) and STD related symptoms (e.g. intermenstrual or post coital blood loss, abdominal pain, more genital discharge than normal).

Rate of CT positives was insufficient in our study to evaluate determinants of CT infection. Therefore, we considered high risk score, defined in accordance with findings from a population based screening, as a proxy for actual high risk. Determinants for high CT risk and CT testing were assessed using univariate and multivariate (using stepwise forward procedure) regression analyses. Determinants for high CT risk were assessed only among sexually active students because students who never had sex are not included in the prediction rule. Interaction terms were assessed between determinants and sex to explore differences between men and women. Since similar results were obtained from analyses adjusted and unadjusted for group (intervention and control), the results presented in this study are unadjusted. Prevalence of CT was calculated with 95 percent confidence intervals (95% CI).

A p-value < 0.05 was considered statistically significant. Analyses were performed with the SPSS package version 14.0 (SPSS, Inc., Chicago, USA).

## Results

Of the 345 participants, 70% (n = 241) were women. Participants' median age was 19 years; with an interquartile range (IQR) of 18-20; only 19 students were older than 22 years (with a maximum of 27 years). Women, on average, were slightly older than men; median age of men was 18 (IQR: 18-20); median age of women was 19 (IQR: 18-20). The majority of the students was of Dutch ethnicity (94% (n = 320)) and lived in urban areas (62% (n = 215)). Of the sexually active participants, 23% (n = 64) had had at least one STD test before (Table 1). Forty nine percent (n = 161) reported regular alcohol use, and 29% (n = 100) used recreational drugs. Men used both alcohol and drugs more often than women (68% (n = 68) and 45% (n = 47), respectively vs. 40% (n = 93) and 22% (n = 53) (p < 0.001)).

**Table 1:** Characteristics and sexual (risk) behavior of 345 vocational school students by sex

	Men (n = 104)		Women (241)		Total (n = 345)		
	%^1^	N	%^1^	N	%^1^	N	
**Sociodemographic**							
Age							
15-19 years	71%	74	63%	152	66%	226	
20-27 years	29%	30	37%	89	35%	119	*
Residence							
Rural	45%	47	34%	83	38%	130	
Urban	55%	57	66%	158	62%	215	
Ethnicity							
Dutch	93%	97	94%	223	94%	320	
Antillean/Surinam	0%	0	1%	2	1%	2	
Turkish/Moroccan	1%	1	3%	7	2%	8	
Other	6%	6	2%	5	3%	11	
**Regular alcohol and drug use** **< 6 months**							
Alcohol	68%	68	40%	93	49%	161	**
Drugs	45%	47	22%	53	29%	100	**
**Sexual behavior**							
Ever sex							
Never sex	17%	17	14%	32	15%	49	
Ever sex	83%	84	86%	204	85%	287	
Life time partners							
1 partner	21%	17	28%	54	26%	71	
2-5 partners	54%	43	57%	111	56%	154	
6 or more	25%	20	16%	31	19%	51	
Sexual debut							
Younger than 16 years	44%	36	45%	88	45%	124	
16 years and older	56%	45	56%	109	55%	154	
Condom use last sexual contact							
No	55%	45	69%	138	65%	183	
Yes	45%	37	31%	61	35%	98	*
Partner change in past 2 months							
No	75%	62	85%	171	82%	233	
Yes	25%	21	15%	30	18%	51	*
Concurrent sexual partners in past (max.) 4 partners							
No	93%	78	97%	196	95%	274	
Yes	7%	6	3%	7	5%	13	
Sex while going out < 6 months							
No	35%	29	25%	49	28%	78	
Yes, with steady partner	44%	36	66%	129	59%	165	
Yes, with casual partner	21%	17	9%	18	13%	35	*
**Types of mixed partnerships**							
by age							
High risk age mixing	75%	61	74%	144	74%	205	
Low risk age mixing	25%	20	26%	51	26%	71	
Sexual orientation							
Opposite sex activities	95%	78	94%	187	95%	265	
Same sex activities	5%	4	6%	11	5%	15	
**Previous STD testing**							
No	77%	64	78%	155	77%	219	
Yes, in < 6 months	8%	7	12%	24	11%	31	
Yes, > 6 months ago	15%	12	11%	21	12%	33	
High CT risk***							
High risk	70%	59	77%	157	75%	216	
Low risk	30%	25	23%	46	25%	71	
**Sexual health**							
Sexual health problems	14%	10	35%	65	29%	256	*
Worried about pregnancy	23%	16	55%	103	46%	256	*
STD related symptoms	6%	5	39%	71	29%	260	*

^1^Missings were excluded from calculation proportions

* = p < 0.05 ** = p < 0.001, indicates differences between men and women

*** based on CT prediction rule (Götz et al, 2005)

All variables below sexual behavior are calculated among sexually active participants

Rates of problems related to sexual health, teenage pregnancy and STD related symptoms were higher for women than for men (all p < 0.05).

Seven percent (n = 16) of women and none of the men reported experience with involuntary sex.

### STD related risk behavior

Eighty five percent (n = 287) of the students reported they ever had sex; most had had two or more partners (n = 205). These participants had been sexually active for a median of 3 years (IQR: 3-5). Sixty five percent (n = 183) had not used a condom at last sexual contact. Men reported condom use more often than women (respectively 45% (n = 37) and 31% (n = 61), p < 0.05). A majority of the students (74%, n = 205) reported risky age mixing i.e. at least one partner older for women and at least one partner younger for men.

Eighteen percent (n = 51) had changed partners in the past two months. Men more often reported recent partner change than women (25% (n = 21) and 15% (n = 30), respectively, p < 0.05). Most students (91%) went to discotheques or bars (men 97%, women 89%, p < 0.05). Students frequently reported they had sex while going out, men more often with casual partners.

### High CT risk and its determinants in sexually active adolescents

Of the sexually active men and women, 75% (n = 216) had high CT risk according to the predefined prediction rule (see methods) (Table 1). In both men and women, concurrency, mixing behavior by age, early sexual debut, drug use, sexual health problems, teenage pregnancy, STD-related symptoms and history of STD testing were univariately associated with having a high risk score (all p < 0.05). Early sexual debut, mixing behavior by age, being worried about teenage pregnancy and a history of STD testing were independently associated with having a high risk of CT (Table 2).

**Table 2:** Determinants for high risk score and actual current Chlamydia trachomatis testing among 287 sexually active vocational school students, South Limburg, The Netherlands 2008/2009

	HIGH RISK SCORE						CT TESTING					
	**OR uni** **variate**	**95%CI**	**P-value**	**OR multi** **variate**	**95%CI**	**P-value**	**OR uni** **variate**	**95%CI**	**P-value**	**OR multi** **variate**	**95%CI**	**P-value**

Female sex	ns						2.08	(1.24-3.49)	.005	ns		
No condom use last contact	na						2.87	(1.72-4.75)	< 0.001	2.94	(1.65-5.24)	< 0.001
Early sexual debut	3.60	(1.91-6.79)	< 0.001	2.99	(1.26-7.12)	.013	ns					
Mixing by age	2.86	(1.58-5.17)	.001	3.53	(1.50-8.30)	.004	ns					
Drug use	2.68	(1.38-5.19)	.004	ns			ns					
Problems related to sexual health	2.22	(1.08-4.55)	.029	ns			2.40	(1.31-4.40)	.005	ns		
Teenage pregnancy	3.15	(1.67-5.95)	< 0.001	2.57	(1.13-5.84)	.025	2.22	(1.32-3.75)	.003	ns		
STD related symptoms	2.24*	(1.10-4.59)	.027	ns			2.73	(1.48-5.04)	.001	2.45	(1.27-4.74)	.007
Previous STD testing	3.99	(1.64-9.72)	.002	3.97	(1.20-13.19)	.024	ns					

*Because specific STD related symptoms (i.e. post coital blood loss in women and frequent urinating in men) were included in the high risk score, these symptoms were excluded

### Current CT testing and its determinants in sexually active adolescents

Of all participants, 53% (n = 183) submitted a self-administered CT test after this was offered to them at their school. Among sexually active students (n = 287), 61% (n = 176) had themselves tested for CT in the current study.

Of the sexually active participants, 22% (n = 64) had a history of STD testing; among the students with high CT risk (n = 216), this rate was 27% (n = 58) (Table 3).

**Table 3:** Previous and current Chlamydia trachomatis testing of 287 sexually active students and 49 not sexually active students

	Self reported previous STD test	Currently tested
Sexually active	22% (64)	61% (176)
Sexually active with high CT risk (n = 216)	27% (58)	65% (141)
Sexually active with low CT risk (n = 71)	9% (6)	49% (35)
Sexually inactive	0% (0)	14% (7)

Among sexually active students 36% (n = 102) were at high risk for CT, had no history of CT testing, but submitted a CT test in the current study. Thirteen percent (n = 37) were at high risk for CT, submitted a test in the current study, and also had a history of CT testing.

Ten percent (n = 29) were at low risk for CT, had no history of CT testing, but submitted a CT test in the current study. Two percent (n = 5) were at low risk for CT, submitted a test in the current study, and also had a history of CT testing.

Sexually active students with a high CT risk submitted a CT test more often (65%, n = 141) than those with a lower risk (49%, n = 35) (OR = 1.93, CI: 1.12-3.33, p < 0.05), but this was mainly true for women (OR = 2.41, CI: 1.35-4.29, p < 0.05) (Table 2). Risk score was not assessed as predictor for testing because all variables, including those comprised in the risk score, were assessed separately.

Independent determinants of CT testing among sexually active students in univariate analysis were female sex, no condom use during last sexual contact, reporting sexual health problems, teenage pregnancy, and STD related symptoms (Table 2). Following multivariate analysis, only STD related symptoms and no condom use during last sexual contact remained in the model (both p < 0.05).

### CT prevalence

Eight participants tested positive for CT; prevalence was 4.5% (n = 8/176) (C.I. 2.3%-8.7%) being 5.2% (n = 7) (C.I. 2.5%-10.3%) in sexually active women (n = 135) and 2.4% (n = 1) (C.I. 0.4%-12.6%) in sexually active men (n = 41) (p = 0.485). Number of CT infections was too low to perform risk factor analyses. All CT infections were found among those with high CT risk. In participants with high risk (n = 140), CT prevalence was 5.7% (n = 8) (C.I. 2.9%-10.9%), being 3.3% (n = 1) (C.I. 0.6%-16.7%) in men (n = 30) and 6.4% (n = 7) (C.I. 3.1%-12.6%) in women (n = 110).

## Discussion

In this study, a relatively simple strategy was used to access high-risk adolescents for sexual health counseling and STD screening. So far, no interventions had been developed for vocational school students in the Netherlands. Our study showed that vocational school students, when CT testing facilities were readily available and appropriate education was provided, were highly likely to have themselves tested for CT; testing uptake increased from 27% (precious self-reported test) to 65% (current test) in those students at highest risk. The overall testing rate of 57% in our study is similar to other comparable school-based studies with a median test rate of 52% (range 33%-68%) [5,7,10,12,14,32]. We consider this a good rate given that in a Dutch screening program, 41% of the people who received a home sampling kit responded by sending in urine and questionnaire [33] and only 16% (in first year) participated in the screening after requesting a CT test package online by sending a sample to the laboratory [24]. Also, coverage and uptake of CT screening in other countries was low, with 22% in 16-24 year olds [34].

School-based screening and treatment programs, e.g. at high schools, appear to be an acceptable and effective strategy to identify infections among at-risk adolescents [12,35,36]. However, whether school based programs targeted at vocational schools are cost-effective remains to be determined. Cost-effectiveness of interventions is an important topic, especially since many countries are exploring screening policies to reduce the number of CT infections in adolescents, such as population-based screening [24]. However, many current programs still are suboptimal in reaching substantial proportions of the population at risk [34,37].

Targeted programs for specific groups at higher risk-especially those who are easy to reach when targeted in the school setting-may be a valuable additional intervention to existing STD prevention and control strategies.

STD control programs are most efficient, i.e., more cost effective, when targeted at high-risk individuals. The increase in testing uptake in the high-risk group, more strongly than in the lower CT-risk group, indicates a well informed self-selection. While this seems mainly true for women, for high-risk men the increase in testing uptake was also high (from 31% ever tested to 53% tested in the current study (data not shown)). When offered CT testing facilities and education, self selection mechanisms in subjects seemed to increase CT testing rate dramatically. Once awareness of CT-associated risks has been heightened, students seem to show an increased tendency to self select for testing, particularly in those who perceive themselves to be at higher risk. A recent study among vocational school students in another Dutch city showed that the intention to undergo testing for STD was very low and could be improved by health promotion interventions aiming to change attitudes, addressing social norms and increasing personal risk perception for STD while also promoting the accessibility of testing facilities [20]. Knowledge about STD also is an important factor in decisions to order and use self-tests [21]. It seems likely that the simple approach taken in our study already covered most of these aforementioned aspects.

In accordance with findings from other national and international studies, vocational school students in our study showed a high risk profile; 75% of the sexually active participants were identified as individuals with high risk of CT. This underlines the assumption that adolescents at vocational schools are an important target population for STD control strategies.

However, the effectiveness of this categorization therefore does not seem particularly strong. Using a cut off score of 8 instead of 6 may increase the effectiveness. This would result in 43% of the sexually active students being defined as individuals with high risk of CT, and CT prevalence in this group would raise to 8.2%. However, with increasing sensitivity, the chance of missing infections would also increase. In this study one of the CT infections would be missed using a cut off score of 8.

Not captured by the high risk algorithm were other characteristics linked to higher STD risk, such as mixing behavior and sexual problems. Partnerships that involve mixing behavior, as well as concurrent partners, have the potential to rapidly accelerate the spread of STD from higher STD prevalence populations to lower STD prevalence populations. Concurrency links individuals into sexual networks, which increases the probability of STD spreading to many members of the network [30]. We considered mixing behavior by age and by sexual orientation, of which mixing by age was most frequent, especially in women who more often have older partners. Gains can be achieved when education offered to vocational school students, is supplemented to address norms, attitude, sexual problems and measures for CT risk which may not be standardly mentioned, such as concurrency of sex partners, mixing behavior and early sexual debut. Mixing behavior and concurrency were, together with early sexual debut, being worried about teenage pregnancy and a history of STD testing, associated with a high risk of CT (p < 0.05), but not with current testing. This means that students are unaware of the relevance of STD testing in view of high-risk behavior, and appropriate attention should be paid to high-risk behavior in prevention programs.

In order to enhance sexual health of adolescents and young adults, it is not sufficient to simply offer STD education and testing facilities to reduce STD. Sexual health is not merely the absence of disease, it also requires a positive and respectful approach to sexuality and sexual relationships, as well as the possibility of having pleasurable and safe sexual experiences [38]. One third of the women in our study had problems with sex which included pain during sex. In addition, 7% of the women reported experience with involuntary sex. Half of the female and 18% of the male students reported problems related to teenage pregnancy.

These sexual health problems were relatively frequently reported in our study, in concordance with national findings [19]. In a nationwide study among young adults under the age of 25, many young adults reported having experienced pain during sex [19] and 18% of the female respondents reported experience with involuntary sex. The findings of our study support the need for professional attention and counseling regarding problems related to sexual health, pregnancy and STD related symptoms in adolescents.

Considering the high frequency of epidemiologically risky behavior reported by the participants and the high CT prevalence found in another Dutch school-based screening [9], STD prevalence of 5.7% in the high-risk group was considered comparable to the first round of the Dutch CT screening program among sexually active high-risk persons applying the same definition for high risk (5.1% in the same study area) [26] and in concordance with the predictive value of the screening criterion of 3.5% [29]. As expected, the prevalence was lower than in the study in Rotterdam [9] which can be explained by the difference in urbanization and the large difference in ethnic populations. Other school-based screening programs in the US demonstrated high CT prevalences (9% to 13% at US high schools) [10,11], while different large US school-based studies showed comparable prevalences [5,7,12-14], e.g., school-based data available from 12 studies with a median CT prevalence of 4.6% in men [13]. Lower prevalences have also been reported, e.g., in an Australian study CT prevalence among high-school students was 1%, and in San Francisco, 2.2% of female students and 0.6% of male students were positive for CT. Most of these studies showed that STD testing was well accepted by adolescents [32,39].

One large study demonstrated that 13.6% of students were reinfected within the same school year [7]. Reinfection was also high (10%) in the Dutch CT screening study [40].

One of the limitations of this study is that the willingness to get tested elsewhere has not been assessed. In 2007, two thirds of STD-related episodes were seen by general practitioners (GPs) and one third by STD clinics [41]. 8% of all consultations of STD clinics concerned attendees aged 15-19 years, and CT prevalence in this group was 17% [2], which is higher than the prevalence in this study. Differences in population characteristics of patients at GPs and STD clinics suggest that each facility serves a different target-group. Clients at STD clinics seem to come more often for a preventive STD-check-up than GP patients with commonly symptomatic STDs [41].

## Conclusions

In view of high rates of sexual activity and risky behavior, school-based sexual health programs for vocational school students may be highly effective in increasing test rates, given their relative ease in accessing and screening large numbers of young adults. Vocational school students are an important target for multifaceted sexual health prevention. Much is to be gained in this group of young adults, characterized by high rates of sexual health problems and STD transmission risk.

## Competing interests

The authors declare that they have no competing interests.

## Authors' contributions

LS performed the statistical analysis and drafted the manuscript. CH participated in the design of the study and helped to draft the manuscript. EB coordinated the acquisition of data and revised the manuscript critically. ND participated in the design of the study, helped with the statistical analysis and helped to draft the manuscript. All authors read and approved the final manuscript.

## Pre-publication history

The pre-publication history for this paper can be accessed here:

http://www.biomedcentral.com/1471-2458/11/750/prepub
